# Short sleep duration associated with the incidence of cardio-cerebral vascular disease: a prospective cohort study in Shanghai, China

**DOI:** 10.1186/s12872-023-03205-y

**Published:** 2023-03-31

**Authors:** Juzhong Ke, Xiaolin Liu, Xiaonan Ruan, Kang Wu, Hua Qiu, Xiaonan Wang, Zhitao Li, Tao Lin

**Affiliations:** Pudong New Area Center for Disease Control and Prevention, Pudong Institute of Preventive Medicine, Fudan University, 3039 Zhangyang Rd, Shanghai, 200136 P. R. China

**Keywords:** Sleep duration, Cardio-cerebral vascular disease, Coronary heart disease, Stroke, Prospective cohort study

## Abstract

**Importance:**

Sleep duration plays an important role in predicting CCVD incidence, and have implications for reducing the burden of CCVD. However, the association between sleep duration and predicted cardio-cerebral vascular diseases (CCVD) risk remains to be fully understood.

**Objective:**

To investigate the effects of sleep duration on the development of CCVD among Chinese community residents.

**Design:**

A prospective cohort study. The baseline survey was conducted from January 2013 to July 2013. The cohort has been followed until December 31, 2016 using a combination of in-person interviews and record linkages with the vital registry of Pudong New Area, Shanghai, China.

**Subjects:**

A total of 8245 Chinese community residents were initially enrolled in the cohort. Of those, 6298 underwent the follow-up examination.

**Exposure:**

Self-reported sleep duration and sleep quality were obtained via the questionnaire. Sleep duration was divided into five categories: ≤5, 6, 7, 8, or ≥ 9 h per day.

**Main Outcome(s) and Measure(s):**

CCVD, Coronary heart disease (CHD) and Stroke occurrence, Hazard ratios (HRs) and 95% confidence intervals (95% CIs) were calculated using Fine-Gray proportional subdistribution hazards models.

**Results:**

During a median follow-up of 3.00 years (IQR 2.92–3.08), we observed 370 participants have had incident CCVD events, of whom 230 had CHDs, 169 had strokes, and 29 had both. After adjustment for relevant confounders, short sleepers (≤ 5 h) had 83% higher risk of total CCVD incidence (HR: 1.83; 95% CI: 1.32–2.54), 82% higher risk of CHD incidence (HR: 1.82; 95% CI: 1.21–2.75), and 82% higher risk of stroke incidence (HR: 1.82; 95% CI: 1.12–2.98) in contrast to the reference group (7 h). Some of these U-shaped relationships varied by age, and were more pronounced in individuals aged < 65 years. Individuals who slept ≤ 5 h per day with baseline hypertension had the highest risk of CCVD incidence (HR: 3.38, 95% CI 2.08–5.48), CHD incidence (HR: 3.11, 95% CI 1.75–5.53), and stroke incidence (HR: 4.33, 95% CI 1.90–9.86), compared with those sleep 7 h and without baseline hypertension.

**Conclusions:**

Short sleep duration is independently associated with greater incidence of CCVD, CHD and stroke.

## Key Points

**Question** What exactly is the relationship between sleep duration and CCVD occurrence in Chinese community residents?

**Findings** This prospective cohort study investigated the effects of sleep duration on the development of CCVD among 6298 Chinese community residents with a median follow-up of 3.00 years. Short sleepers (≤ 5 h) had higher risk of total CCVD, CHD and stroke incidence compared to the reference group (7 h). Some of these U-shaped relationships varied by age, and were more pronounced in individuals aged < 65 years. Individuals who slept ≤ 5 h per day with baseline hypertension had the highest risk of CCVD, CHD and stroke incidence.

**Meanings** Short sleep duration is independently associated with greater incidence of CCVD, CHD and stroke. Preventative strategies are needed to reduce the disease burden of CCVD associated with inadequate sleep duration.

## Introduction

Cardio-cerebral vascular diseases (CCVD) are serious threats to human’s health with high incidence, morbidity, mortality, and many complications [1]. Given the severe consequences and accompanying economic losses, recognition of risk factors may play an essential role in preventing CCVD. Extreme sleep duration has long been considered as an important contributing factor to the risk of CCVD [2–4]. Increasing attention has been paid to explore the sleep duration problems and their effects on various health outcomes including CCVD, diabetes mellitus, and cancer [5, 6].Previous study suggests that between 1985 and 2012, the mean sleep duration has steadily decreased, and the prevalence of short sleep duration (defined as < 7 h) has increased from 22.3 to 29.2% [7]. It’s critical to understand the role of sleep duration in predicting CCVD incidence, since they may have implications for reducing the burden of CCVD.

Several studies investigated the association between sleep duration and the risk of CCVD outcomes, however, uncertainty still exists about the association and the dose-response relationship [8–11]. In addition, whether the relationship between extreme sleep duration and incident CCVD varies by age or sex remains unclear. If combined effects of sleep duration and hypertension exists on CCVD incidence, increased efforts for their identification and treatment should be undertaken. Although a number of studies have examined such relationships, most of them are cross-sectional designed. In addition, few studies have been done in the Chinese population, where disease burden from CCVD is increasing rapidly in the past decades [12]. Here, we carried out a prospective study to illustrate the association between sleep duration and CCVD incidence among a Chinese population, and to investigate whether this association is age/sex specific and/or modified by hypertension status.

## Materials and methods

### Study population

**Fig. 1:**
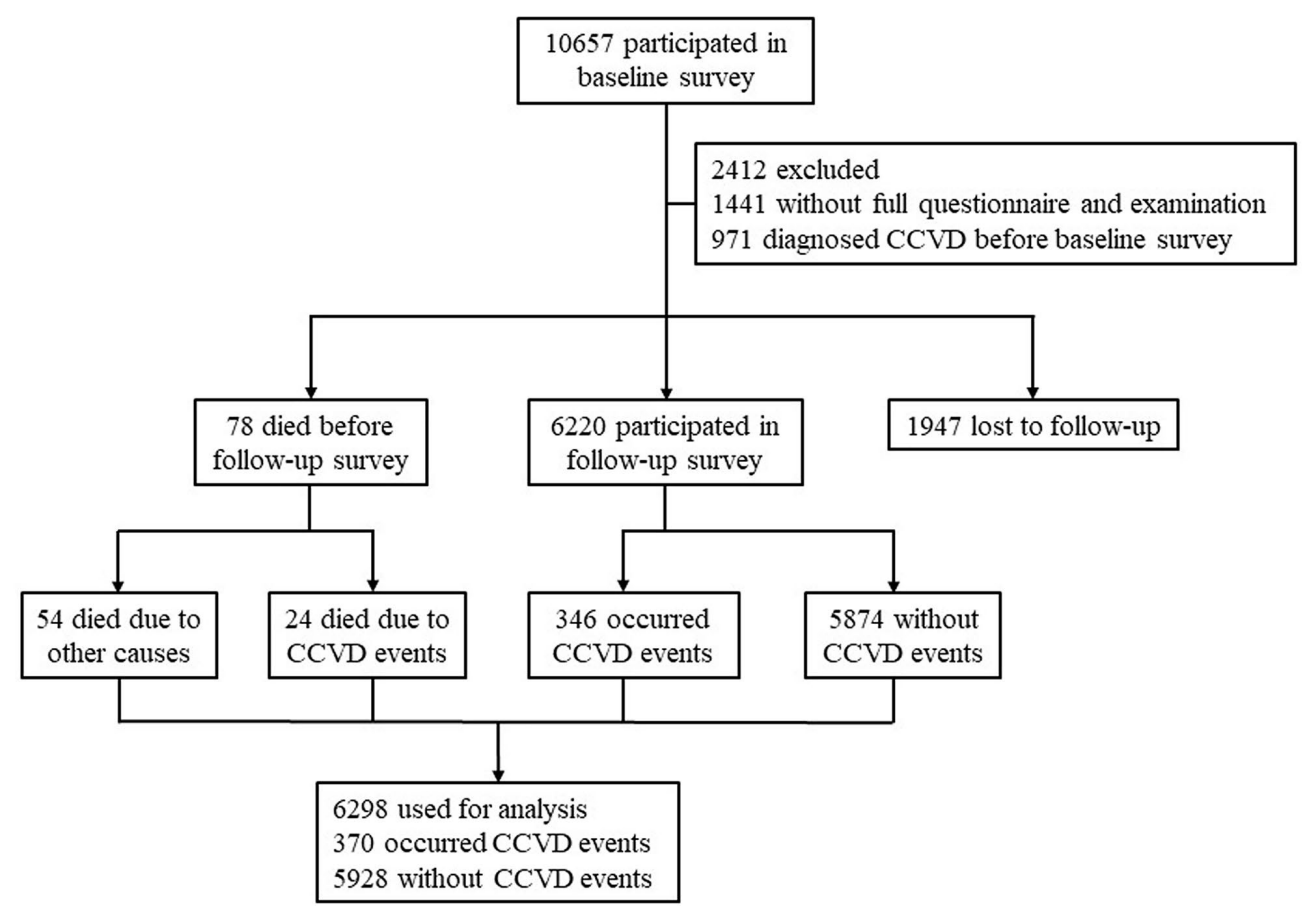
Diagram of this study

This study was performed among subjects of the ongoing prospective population-based Cohort Study in Pudong New Area, Shanghai, China. The study is the sequential research of the previous cross-sectional study [13]. The baseline survey was conducted from January 2013 to July 2013. Of 10,657 subjects in baseline, 1441 were excluded due to the incompletion of the full questionnaire or examination. 971 subjects were excluded because they had been diagnosed CCVD before baseline survey. The remaining 8245 subjects entered the follow-up cohort. 6220 subjects participated the in-person follow-up survey conducted from January 2016 to May 2016. 78 subjects died before the follow-up surveys, of them 24 died due to CCVD events and 54 due to other causes. 1947 subjects were lost to follow up. Altogether, definite CCVD outcomes of 6298 (76.39%) subjects were obtained, with a median follow-up period of 3.00 years (Fig. 1). The study had obtained approval from the Ethical Committee of Center for Disease Control and Prevention of Pudong New Area. Written informed consents were obtained from all subjects.

### Assessment of sleep duration and sleep quality

Self-reported sleep duration and sleep quality were obtained via the questionnaire. Sleep duration was obtained by the question “In the past year, on average, how many hours of sleep do you get in a 24 h period?”, answers were divided into five categories: ≤5, 6, 7, 8, or ≥ 9 h. Sleep quality was also inquired. Difficulty in falling asleep was assessed with the question, “In the past year, how often did you have trouble in falling asleep?” Difficulty in staying asleep with the question, “In the past year, how often did you wake up in the midnight and feel difficult to fall asleep again?” Early morning awakening with the question, “In the past year, how often did you wake up too early in the morning?” Usage of hypnotics with the question, “In the past year, how often did you use hypnotics or other drugs to help you falling asleep?” Patients with responses of “sixteen to thirty times a month” of at least one of the four items were classified as having poor sleep quality [14].

### Assessment of covariates

Information on sociodemographic characteristics (including age, sex, and marital status), smoking, physical activity, alcohol and tea consumption were collected through a questionnaire by face-to-face interview with trained interviewers. Smoking was classified as current smoker (with at least one cigarette a day for at least the previous 6 month) or not. Physical activity was classified as regular exerciser (participating in sports activities for at least once per week in the previous 5 years) or not. Alcohol consumption and tea consumption were classified as regular drinker (at least three times per week for at least the previous 6 month) or not. Standard procedures were used to measure height, weight, SBP and DBP. Body mass index (BMI) was calculated as weight (kg)/height (m [2]). Hypertension was defined as SBP ≥ 140 mmHg or DBP ≥ 90 mmHg, or self-reported history of hypertension. Type 2 diabetes referred to self-reported history of type 2 diabetes, or elevated fast plasma glucose (≥ 7.0mmol/L), or elevated 2-hours plasma glucose (≥ 11.1mmol/L) during 75-g oral glucose tolerance test. Dyslipidemia was defined as LDL-C ≥ 4.13mmol/L, or TC ≥ 6.20mmol/L, or HDL-C < 1.03mmol/L, or TG ≥ 2.26mmol/L, or self-reported history of dyslipidemia. The serum C-reactive protein (CRP) levels were detected by particle enhanced immunonephelometric method.

### Outcomes

The primary outcome in the current study was CCVD incidence, defined as a physician diagnosis or death of coronary heart disease (CHD) or stroke that occurred in the follow-up duration. Subjects were defined as having CHD if they had angina, myocardial infarction, silent myocardial ischemia or ischemic heart failure, and as having stroke if they had ischemic or hemorrhagic stroke. Deaths were identified through records linking with the vital registry of Pudong New Area, Shanghai, China. Underlying death causes were coded according to ICD-10: I21-I25 for CHD deaths, I60-I64 for stroke deaths. Both CHD and stroke were considered CCVD.

### Statistical analysis

Statistical analyses were performed using SPSS 22.0 and SAS 9.4. Subjects were classified into five groups based on sleep duration. Chi-square and one-way ANOVA tests were used to compare the categorical and numeric variables among groups. Taking competing-risks into consideration, Fine-Gray proportional subdistribution hazards model was performed to evaluate the associations of sleep duration with the risk of incident CCVD, covariates were choose based on evidence from literature. Potential confounders included age, sex, marital status, current smoking, alcohol consumption, tea consumption, physical activity, hypertension, type 2 diabetes, dyslipidemia, BMI, CRP, and sleep quality. nonlinear trends of incident CCVD risk were tested by restricted cubic spline Cox regression using 3 knots placed at the 5th, 50th, and 95th percentiles of sleep duration, with sleep duration of 7 h as the reference group [15]. We analyzed the potential interactions by adding interaction terms of age and sex with sleep duration, respectively. We conducted subgroup analyses separately by age group (above and below 65 years). Combined effects of sleep duration and hypertension on the risk of incident CCVD were also evaluated, taking sleep duration of 7 h and non-hypertension as the reference category. The results were presented as hazards ratios (HRs) with 95% confidence intervals (CIs), *p* < 0.05 were considered statistically significant.

## Results

### Baseline characteristics

Baseline characteristics of subjects are presented in Table 1. Of the 6298 subjects in the study population, 682 subjects (10.83%) reported sleep duration ≤ 5 h/day, 1282 (20.36%) slept 6 h/day, 1522 (24.17%) slept 7 h/day, 2185 (34.69%) slept 8 h/day, and 627 (9.96%) slept ≥ 9 h/day. Compared to those who slept 7 h a day, those with shorter sleep duration were more likely to be older, female and unmarried. Shorter sleepers were more likely to be diagnosed with hypertension, type 2 diabetes, dyslipidemia and suffered poorer sleep quality; and less likely to be current smoker or tea drinker.

### Association between sleep duration and CCVD incidence

**Fig. 2:**
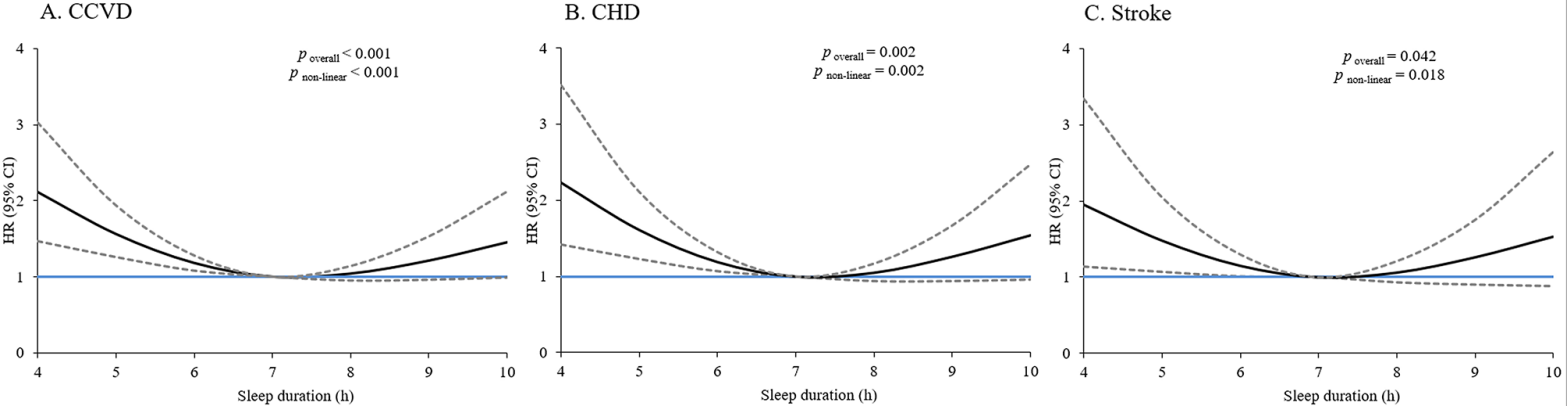
Multivariable adjusted spline curves for association between sleep duration and risk for (A) CCVD incidence, (B) CHD incidence, and (C) stroke incidence, adjusted for age, sex, marital status, current smoking, alcohol consumption, tea consumption, physical activity, hypertension, type 2 diabetes, dyslipidemia, BMI, CRP, and sleep quality

During a median follow-up of 3.00 years (IQR 2.92–3.08), we observed 370 participants have had incident CCVD events, of whom 230 had CHDs, 169 had strokes, and 29 had both. 24 individuals died due to fatal CCVD events, including 16 CHDs and 8 strokes. After adjustment for relevant confounders, short sleepers (≤ 5 h) had 83% higher risk of total CCVD incidence (HR: 1.83; 95% CI: 1.32–2.54), 82% higher risk of CHD incidence (HR: 1.82; 95% CI: 1.21–2.75), and 82% higher risk of stroke incidence (HR: 1.82; 95% CI: 1.12–2.98) in contrast to the reference group (7 h). Although both short and long sleepers tended to have a higher risk of CHD incidence, long sleep duration (≥ 9 h) was not associated with total CCVD incidence (HR: 1.20; 95% CI 0.82–1.75), CHD incidence (HR: 1.37; 95% CI 0.86–2.17), or stroke incidence (HR: 1.15; 95% CI 0.65–2.04) (Table 2). The nonlinear associations of sleep duration with risk of incident CCVD were illustrated by the restricted multivariable cubic spline plots as shown in Fig. 2. Multivariable spline analyses showed U-shaped trends in CCVD, CHD and stroke incidence with bottom point at 7 h. Compared to subjects with 7 h sleep duration, shorter sleepers were at higher risk of CCVD, CHD and stroke incidence (*p* < 0.001, = 0.002, 0.042, respectively).

### Age-specific association between sleep duration and CCVD incidence

**Fig. 3:**
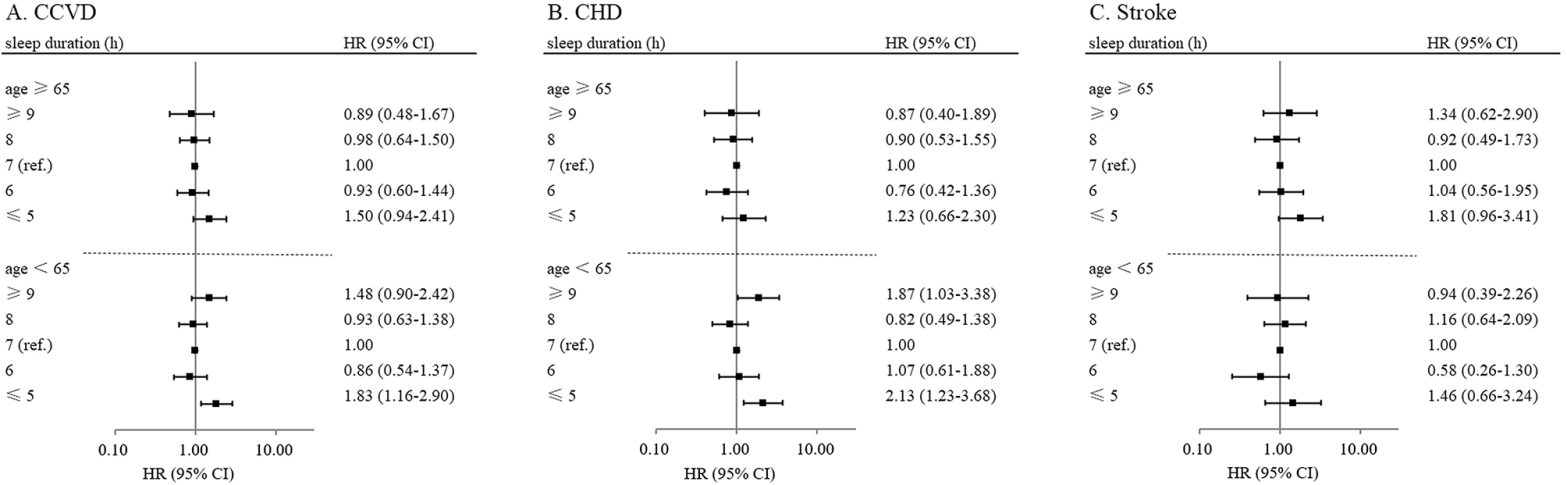
Hazard ratios (95% CI) for incident CCVD(A), CHD(B), and Stroke(C) in subjects above and below 65 years according to sleep duration. The adjusted covariates included age, sex, marital status, current smoking, alcohol consumption, tea consumption, physical activity, hypertension, type 2 diabetes, dyslipidemia, BMI, CRP, and sleep quality, with sleep duration of 7 h as the reference group

In stratification and interaction analysis, the U-shaped relationship were more prominent among young and middle-aged individuals. In the subgroup of participants aged less than 65 years old, compared with those who slept 7 h per day, those slept ≤ 5 h had 2.13 times more risk of incident CHD (HR: 2.13; 95% CI 1.23–3.68), and those slept ≥ 9 h had 87% increased risk of incident CHD (HR: 1.87; 95% CI 1.03–3.38), whereas the U-shaped relationship was not found in the subgroup of participants aged more than 65 years old (*p* for interaction = 0.032). The results were similar for CCVD incidence, but no significant interaction was found (Fig. 3). The interactions between sleep duration and sex were also analyzed, no significant interaction was observed (all *p* for interaction > 0.05).

### Combined effects of sleep duration and hypertension on CCVD incidence

**Fig. 4:**
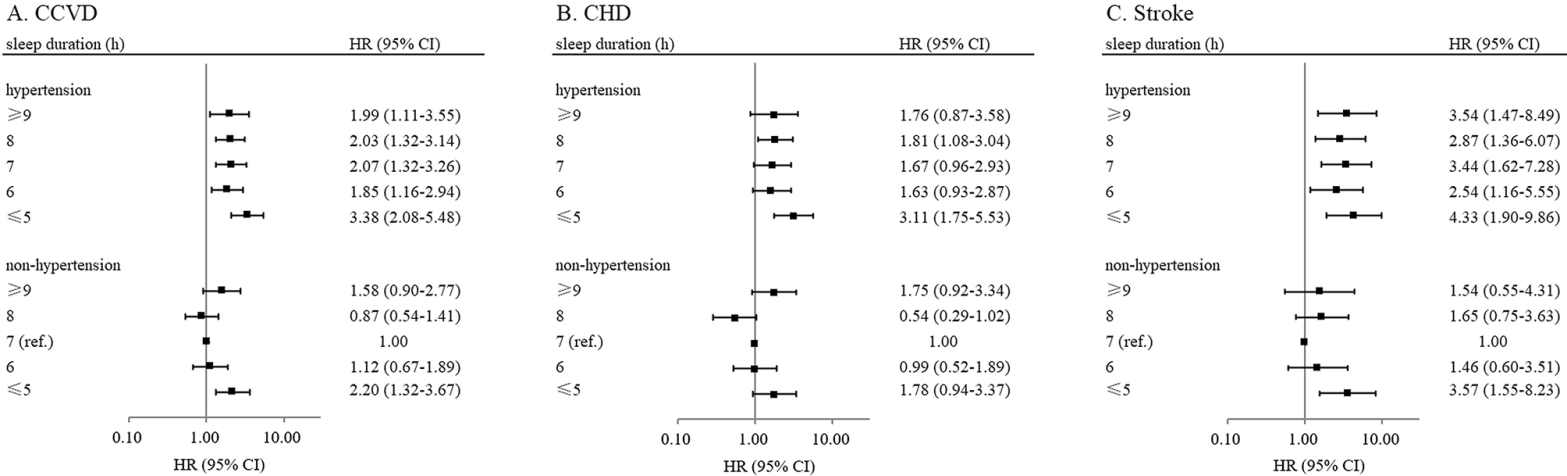
Joint association of sleep duration and baseline hypertension status with (A) CCVD incidence, (B) CHD incidence, and (C) stroke incidence. All HRs and 95% CIs were computed with sleep duration of 7 h and absence of hypertension as the reference group, adjusted for age, sex, marital status, current smoking, alcohol consumption, tea consumption, physical activity, hypertension, type 2 diabetes, dyslipidemia, BMI, CRP, and sleep quality

In this study, individuals with hypertension had significantly elevated risk of CCVD incidence (HR: 1.67, 95% CI 1.35–2.07), CHD incidence (HR: 1.59, 95% CI 1.21–2.08), and stroke incidence (HR: 1.93, 95% CI 1.40–2.65), relative to non-hypertension individuals. We further analyzed the combined effects of sleep duration and hypertension on CCVD incidence. Individuals who slept ≤ 5 h per day with hypertension had the highest risk of CCVD incidence (HR: 3.38, 95% CI 2.08–5.48), CHD incidence (HR: 3.11, 95% CI 1.75–5.53), and stroke incidence (HR: 4.33, 95% CI 1.90–9.86), compared with those sleep 7 h and without hypertension. Among non-hypertension individuals, elevated risk of CCVD incidence and stroke incidence was observed in ≤ 5 h sleepers, whereas risk of CHD incidence in ≤ 5 h sleepers was elevated but not statistically significant (Fig. 4).

## Discussion

In this prospective cohort study, we found that after controlling for a variety of potential confounders, short sleepers (≤ 5 h per day) had higher risk of CCVD, CHD and stroke incidence, and the U-shaped relationship appeared to be seen mainly in individuals less than 65 years old. In addition, there were combined effects of sleep duration and hypertension on incident CCVD risk. This study indicated that maintain a moderate sleep duration may be considered as an effective intervention strategy to prevent CCVD. The study population was randomly recruited from community residents living in Pudong New Area. Pudong New Area has a resident population of about 5 million, accounting for 20% of the total population in Shanghai. In addition, Pudong New Area is the only district with both urban and rural populations in Shanghai, with diverse socioeconomic status, which is a representative model for other populations [16]. Thus, the findings of this study might provide evidence to optimize CCVD control strategy.

This study explored the associations of short and long sleep duration with CCVD incidence based on a Chinese community population, with longitudinal design and multiple potential confounders taken into consideration. In accordance with previous studies, this study adds to the evidence for a U-shaped association of sleep duration with CCVD incidence, and found a robust association between short sleep and increased CCVD risk [2, 6, 17–19]. However, the effects of long sleep duration remain unclear, although some studies reported that sleep duration longer than 8 h/d was associated with an increased risk of CCVD [2, 19, 20], we failed to reach statistical significance in the long sleep group, the result of which was similar to a recent study conducted in Taiwan population [17]. Explanation of the controversial results might be attributed to the diverse races, ages, and lifestyles across studies.

We observed that the increased risk with extreme sleep duration was more prominent in young and middle-aged individuals. This age-specific trend is in line with results from a previous study conducted in Australian adults, which showed that short sleep was associated significantly with increased prevalence of heart disease in middle-aged and elderly adults, but not in the very elderly [21]. A study conducted in British population suggested that the increased risk of stroke with short sleep duration was more prominent among middle-aged subjects, but with long sleep more pronounced among the elderly adults [22]. Similarly, the association between sleep duration and hypertension varies by age, Fang J, et al. showed that short sleep was associated with higher likelihood of hypertension among middle-aged adults, but did not among the elderly adults [23]. Although the underlying mechanisms remains unclear, sleep duration might have different implications in different age groups, the increase of age may cover up the influence of sleep duration on CVD incidence among older individuals and leave the detrimental effects robust in younger adults. The interaction by age needs to be tested by larger studies in the future.

Hypertension is the most important risk factor for CCVD, the severe outcome of hypertension is primarily due to its driving role in the development of CCVD [24]. Lower sleeping times are associated with an increased risk of subclinical atherosclerosis, which could be induced by hypertension [25, 26]. Meanwhile, as a chronic disease hallmarked by chronic inflammation, atherosclerosis development has been shown to be potential mechanisms for CCVD development [27]. In this study, the greatest risk of CCVD incidence was found in individuals with both hypertension and short sleep duration, suggesting that appropriate sleep duration is particularly important for patients with hypertension.

As a potentially important confounder, sleep quality has been found to moderate the association between sleep duration and cardiovascular outcomes [28]. Even after excluding subjects with cardiovascular diseases and depression, symptoms of poor sleep quality such as difficulty initiating sleep and non-restorative sleep are associated with a modestly higher risk of CCVD mortality [29].Therefore, when we investigating the linkage of sleep duration with increased CCVD risk, effects of sleep quality should be ruled out. It is noteworthy that the role of individual differences and their preferences in terms of sleep habits should be taken into consideration [18]. Short sleep duration is often reported in conjunction with lower socioeconomic status and depressive mood, which could bias the link between short sleep and cardiovascular risk [30, 31]. High-risk behaviors such as smoking, drinking, and low physical activity are also prevalent in short sleepers [23]. Adjusting for these established CCVD risk factors did not considerably change the estimated association in this study.

Sleep duration may be involved in the pathogenesis of CCVD, as numerous studies have suggested the relationship between sleep duration and obesity, diabetes as well as increased levels of CRP [32–34]. The mechanisms that underlie these associations are still under investigation. Short sleep duration may lead to sympathetic nervous system overactivity and changes in circadian rhythm, resulting in the rise of blood pressure and arterial stiffness, which may promote cardiovascular risk [28, 35]. Besides, sleep loss impairs glucose homeostasis and insulin sensitivity, leads to health outcomes related to metabolic systems, such as obesity and type 2 diabetes, both plays an important role in development of CCVD [36, 37]. In addition, sleep deprivation has been reported to be associated with elevated levels of inflammatory cytokines, such as CRP and interleukin-6, which is likely to increase CCVD risk through damaging the body’s immune function [34]. Proteinuria marks an increased risk of renal damage related to CCVD. Results from observational studies revealed that short sleep duration is associated with prevalence of proteinuria in a dose-dependent manner [38, 39]. In patients with chronic kidney disease, short sleep duration is associated with faster decline in renal function and progression to end stage renal disease [40, 41]. Thus, proteinuria might also intermediate the association between short sleep duration and CVD. Further research is needed to clarify these potential roles.

The strengths of this study are its prospective design, relatively large sample size, and the adjustment of many potential risk factors. The findings of this study may provide some new insight into CCVD prediction and prevention. This study has several limitations. First, information about sleep duration were obtained through questionnaires. Sleep duration was a kind of lifestyle factor that might be affected by many factors, thus exposure misclassification bias and discrepancy between measures of subjective sleep duration and objective sleep duration might exist. Previous study suggested that objective sleep duration derived from actigraphy was significantly correlated with subjective self-report sleep duration, but was about 0.5-1 h shorter than the subjective one [42]. Therefore, we may have underestimated the health implications of short sleep duration. Second, the follow-up period of this study was relatively short. Third, although a range of potential confounders were included in the analysis, we could not rule out the possibility of residual/unmeasured confounding.

## Conclusions

Short sleep duration is associated with greater incidence of CCVD, independent of other traditional CCVD risk factors. This association appeared to be seen mainly in individuals less than 65 years old. The strongest association with CCVD incidence was found in short sleepers with hypertension. Implementation of preventative strategies may be needed to reduce the disease burden of CCVD associated with inadequate sleep duration.

**Table 1:** Baseline characteristics stratified by sleep duration

	Sleep duration (h)					Total (n = 6298)	*p*
	≤5	6	7	8	≥ 9		
	(n = 682)	(n = 1282)	(n = 1522)	(n = 2185)	(n = 627)		
Age (years)	62.01 (9.68)	60.77 (10.50)	57.65 (11.44)	56.26 (12.01)	57.42 (12.06)	58.25 (11.53)	<0.001
Male (%)	204 (29.91%)	435 (33.93%)	583 (38.3%)	879 (40.23%)	233 (37.16%)	2334 (37.06%)	<0.001
Married (%)	595 (87.24%)	1134 (88.46%)	1383 (90.87%)	1975 (90.39%)	556 (88.68%)	5643 (89.60%)	<0.001
Current smoking (%)	92 (13.49%)	185 (14.43%)	276 (18.13%)	416 (19.04%)	123 (19.62%)	1092 (17.34%)	<0.001
Alcohol consumption (%)	80 (11.73%)	137 (10.69%)	185 (12.16%)	296 (13.55%)	80 (12.76%)	778 (12.35%)	0.161
Tea consumption (%)	138 (20.23%)	329 (25.66%)	411 (27.00%)	630 (28.83%)	183 (29.19%)	1691 (26.85%)	<0.001
Physical activity (%)	189 (27.71%)	367 (28.63%)	400 (26.28%)	557 (25.49%)	165 (26.32%)	1678 (26.64%)	0.331
Hypertension (%)	304 (44.57%)	595 (46.41%)	619 (40.67%)	865 (39.59%)	243 (38.76%)	2626 (41.70%)	<0.001
Type 2 diabetes (%)	150 (21.99%)	276 (21.53%)	287 (18.86%)	390 (17.85%)	129 (20.57%)	1232 (19.56%)	0.030
Dyslipidemia (%)	365 (53.52%)	617 (48.13%)	666 (43.76%)	1007 (46.09%)	309 (49.28%)	2964 (47.06%)	<0.001
Poor sleep quality (%)	99 (14.52%)	57 (4.45%)	24 (1.58%)	30 (1.37%)	13 (2.07%)	223 (3.54%)	<0.001
BMI (kg/m^2^)	24.92 (3.31)	25.12 (3.44)	25.18 (4.61)	25.04 (3.41)	24.94 (3.66)	25.07 (3.75)	0.487
CRP (mg/L)	0.27 (0.07, 0.93)	0.25 (0.07, 0.90)	0.23 (0.07, 0.87)	0.24 (0.07, 0.94)	0.27 (0.07, 1.01)	0.25 (0.07, 0.94)	0.611

Data are n (%), mean (SD) or median (IQR).

BMI: body mass index, CRP: C-reactive protein

**Table 2:** Association between sleep duration and cardiovascular disease incidence in cohort participants

Sleep duration (h)	Person-years	Incident cases	Incidence (per 1000 person-years; 95% CI)	Hazard ratio (95% CI)	*p*
CCVD					
≤5	1949	73	37.45 (29.02–45.88)	1.83 (1.32–2.54)	< 0.001
6	3748	74	19.74 (15.29–24.20)	0.97 (0.71–1.33)	0.848
7 (ref.)	4470	81	18.12 (14.21–22.03)	1.00	
8	6440	103	15.99 (12.93–19.06)	0.94 (0.70–1.25)	0.657
≥ 9	1840	39	21.19 (14.61–27.77)	1.20 (0.82–1.75)	0.357
CHD					
≤5	1996	46	23.05 (16.46–29.63)	1.82 (1.21–2.75)	0.004
6	3786	46	12.15 (8.66–15.64)	0.98 (0.65–1.46)	0.910
7 (ref.)	4516	51	11.29 (8.21–14.38)	1.00	
8	6518	59	9.05 (6.75–11.35)	0.84 (0.58–1.22)	0.368
≥ 9	1866	28	15.01 (9.49–20.53)	1.37 (0.86–2.17)	0.185
Stroke					
≤5	2012	34	16.9 (11.27–22.53)	1.82 (1.12–2.98)	0.017
6	3817	32	8.38 (5.49–11.28)	0.91 (0.57–1.47)	0.708
7 (ref.)	4538	36	7.93 (5.35–10.51)	1.00	
8	6508	50	7.68 (5.56–9.80)	1.04 (0.68–1.60)	0.847
≥ 9	1871	17	9.09 (4.79–13.39)	1.15 (0.65–2.04)	0.629

Hazard ratios and 95% CIs are adjusted for age, sex, marriage status, smoking status, drinking status, tea consumption, physical activity, hypertension, diabetes, dyslipidemia, BMI, CRP and sleep quality

## Data Availability

All data generated or analyzed during this study are included in this published article.
